# Some Account of the More Important Medical Societies Now Existing in London

**Published:** 1900-10-06

**Authors:** D'Arcy Power


### Some Account of the More Important Medical Societies now Existing in London.

By D'Arcy Power, M.B.Oxon., F.R.C.S.

The amelioration of manners and the literary taste which prevailed during the reign of Queen Anne and under the sway of the early Georges paved the way for the formation of societies and clubs in every condition of life. But the proud position held by the physicians, coupled with the illiteracy of the surgeons and the humbleness of the apothecaries, wholly prevented the various members of the medical profession from meeting on any common ground of knowledge or friendship. The Royal Society long supplied the wants of the more learned in our profession, and if the older volumes of its *Transactions* be examined they will be found to contain innumerable isolated cases of exactly the same kind as those which are brought before the medical societies now in existence.

During the latter half of the last century there arose in London a succession of great teachers who were able to foster, or, as is more likely, were the direct outcome of, a desire on the part of the rank and file of the profession to receive, a better education than had hitherto been provided. Foremost amongst these men were William and John Hunter, who had a genius for so drilling their pupils as to make them part of a complex machine for the advancement of medical knowledge. In pursuance of this design numerous medical societies came into existence, some of which were modelled upon the medical societies of Edinburgh, themselves copied from the societies at Padua and Leyden. The early medical societies of London were either connected with an individual school or they were founded for the interchange of ideas with men of different schools. A few of the first class still remain, for the Middlesex Hospital Medical Society was established in 1774, and the Abernethian Society held its first meeting at St. Bartholomew's Hospital in 1795 under the name of the Medical and Philosophical Society. The Physical Society of the United Borough Hospitals, founded in 1771, only died in 1852, but left vigorous offspring in the Pupils' Physical Society at Guy's Hospital and the Medical and Physical Society at St. Thomas's Hospital.

### THE MEDICAL SOCIETY OF LONDON.

The Medical Society is the solitary survivor of the general medical societies founded in the last century. It owes its formation to the energy of Dr. John Coakley Lettsom, the successful physician, of whom it was said:—

> When any sick to me apply,
> I physick, bleed and sweats 'em:
> If after that they choose to die,
> What's that to me,——I. Lettsom.

The epigram, however, is absolutely pointless as applied to Lettsom, the possessor of all the Quaker virtues, who freed the slaves on his ancestral estate when he had no practice and their emancipation seemed to promise him ruin. The first meeting of the Medical Society was held on Wednesday, May 19th, 1773, when Lettsom was appointed treasurer and Edward Ford secretary. It was agreed that the meeting room should be "as nearly central to the respective members as possible," and the Queen's Arms Tavern in Newgate Street was suggested as a fitting place, the hire of the room not to exceed ten guineas a year. The Queen's Arms business fell through, and at the next meeting Lettsom was asked to secure a vacant room in Wardrobe Court, and was authorised to expend £15 a year on the rent. Even this sum failed to procure sufficient accommodation, and the Society continued to meet on the first floor of a house in Crane Court, Fleet Street, then occupied by a person called Pearce. The purpose of the Society was "to give the practitioners of the healing art frequent opportunities of meeting together and conferring with each other concerning any difficult or uncommon cases which may have occurred, or communicating any new discoveries in medicine which have been made either at home or abroad." It was decided at the sixth meeting of the Society that the present number of its members should be limited to thirty physicians, thirty surgeons and thirty apothecaries, three standing committees of reference being at the same time appointed—one of three physicians, one of three surgeons, and one of three apothecaries. Within two months of its foundation Lettsom laid the foundation of the present library by presenting *Percivall's Medical Observations* and *The Medicinal Education of Children*.

Troubles soon arose, for as early as the autumn of 1774 considerable unrest occurred within the Society in connection with the framing of the rules, and matters rose to such a crisis that on January 18th, 1775, when the members repaired to Crane Court to hold the anniversary meeting they found the doors of their room "unwarrantably locked," and that the president and his two secretaries had resigned their offices. Much diplomacy resulted from this lockout, and it was not until March 7th, 1775, that the new president, Lettsom, was able to "send a message to all the members of the Society acquainting them that harmony is restored and requesting their attendance as usual." It is pleasant to observe that the trouble led to no lasting ill-effects, for Millar, the rebel leader, was invited to deliver the annual oration in the following year. In 1788 the Society moved to Bolt Court, Fleet Street, Dr. Lettsom's house, which he bequeathed to the Society so long as its members did not fall below ten. The Society was again nearly wrecked in 1784 by violent party disputes over the candidature of two of its members, Dr. Cooke and Dr. Whitehead, for an appointment at the London Hospital. At this time Dr. Whitehead—formerly a linendraper who failed at Bristol —was a Quaker, and by the Friends' interest he secured the appointment. But shortly afterwards he seceded to John Wesley, whom he attended in his last illness and whose funeral sermon he preached. The result of these squabbles was very disastrous, and the meetings became very small, for sometimes no more than four members attended. Dr. James Sims, too, was re-elected president time after time, and it was only when he had served the office for twenty-two years that he was displaced by the strenuous exertions of the younger Fellows. Sims had a valuable collection of books, which he made over to the Medical Society in consideration of an annuity of £30 a year to be paid to himself and his wife, and of £45 annually to the survivor; the annuity to commence in 1802. Dr. Sims seems to have been quite a character, for it is said of him that "he was a good-humoured, pleasant man, full of anecdote, an ample reservoir of good things, and for figures and facts a perfect chronicle of other times. He had a most retentive memory, but when that failed he referred to a book of knowledge from which he quoted with oracular authority." He was an Irishman, born in 1741, and a friend of Lettsom. He made his fortune and retired to Bath in 1810, where he died ten years later. The repeated re-election of Dr. Sims led to a secession of many of the Fellows, who founded the (Royal) Medical and Chirurgical Society in 1805. Lettsom, Clutterbuck, Hancock, Pinckard and a few other men of eminence remained staunch, and the original society proceeded on its way, only to be disturbed by fresh dissensions when Thomas Joseph Pettigrew was elected secretary in opposition to Dr. Birkbeck, who was many years his senior. Lettsom continued to guide the Society, and by his influence the Fothergillian gold medal was founded in memory of his guardian and benefactor, John Fothergill [1712-1780]. The fund was afterwards permanently endowed by Dr. Anthony Fothergill [*d.* 1813], who does not appear to have been in any way related to his namesake. Lettsom died on November 1st, 1815. The Society continued to meet in his house at Bolt Court, Fleet Street, until 1850, when it was found more convenient to move, as the value of property in the City of London was rising steadily. It was found, too, that the number of members had fallen to 60, and it was decided to amalgamate with the Westminster Medical Society, and to take new premises in George Street, Hanover Square. The joint society remained here for twenty-one years, and at the expiration of the lease removed to the quarters which it now occupies in Chandos Street, Cavendish Square. Of late years its finances have been managed with such care and the influx of members has been so great that in spite of its age it is one of the largest and most prosperous medical societies in London.

### THE WESTMINSTER MEDICAL SOCIETY.

The Westminster Society still exists in virtue of amalgamation with the Medical Society, though its name has disappeared, because the property of the Medical Society is so held that it could not change its name, whilst the Westminster Society was free from any incumbrance of worldly possessions. The Westminster Society was founded by Sir C. Mansfield Clarke and Sir Benjamin Brodie in 1809. It was chiefly recruited from the Great Windmill Street School of Medicine, and the payment of a guinea constituted each student a member for life. The meetings were held in the museum of the school which had been built and filled by William Hunter, and in 1824 it is said that 1,000 names were on the roll of membership. By the death of Wilson in 1851 the whole responsibility of the school was thrown upon (Sir) Charles Bell, and when Bell was placed at the head of the medical department of the newly-founded London University, now University College, in 1828, the school rapidly dwindled until it became extinct about 1836. The fortunes of the Westminster Medical Society waned with those of the Hunterian School. It met first in Sackville Street, then in Windmill Street again, afterwards in Exeter Hall, and last of all in Savile Row. In 1843 it consisted of about twelve members, but it was resuscitated, and at the time of its amalgamation with the Medical Society there were 237 names upon its books. The Society's work is worthy of recognition, for it took the lead in discussing the nature and treatment of cholera during the epidemic of 1832, and by its discussions upon the Anatomy Bill it did much to lessen the popular prejudice against dissection.

### THE ROYAL MEDICAL AND CHIRURGICAL SOCIETY OF LONDON.

This Society, which is justly looked upon as the premier medical society of England, was founded, as has been said, by members who seceded from the Medical Society in consequence of disagreements arising out of Dr. Sims' long tenure of the office of president. The inaugural meeting was held at the Freemasons' Tavern on May 22nd, 1805, Dr. William Saunders, F.R.S., physician extraordinary to the Prince of Wales, being voted into the chair. It was determined that a society should be formed comprehending the several branches of the medical profession, for the purpose of conversation on professional subjects, for the reception of communications and for the formation of a library. The Society was to be called the Medical and Chirurgical Society of London. Its meetings were to be held in some central situation, and its affairs were to be conducted by a president, four vice-presidents, a treasurer, three secretaries (one of whom was to be foreign secretary), and a certain number of members, who together were to form a Council, to be elected annually. No gentleman was to be eligible to the office of president or vice-president for more than two years in succession. The admission fee was to be six guineas, and the annual subscription three guineas. Twenty-six names were at once entered for membership, and Dr. Yelloly, physician to the London Hospital, was requested to act as secretary of a temporary committee to arrange further details. The first meeting of the Society was held at 2 Verulam Buildings, Gray's Inn, in December, 1805, and such eminent members of the profession as Abernethy, Aikin, Babington, Baillie, Bateman, Blaine, Blizard, Astley Cooper, Jenner, Humphry Davy, James Wilson, Daniel Rutherford, and Alexander Marcet enrolled themselves Fellows. John Sims himself joined the Society, but it was not until 1809 that he was elected a member of the Council. The first volume of the *Transactions* appeared in 1809, and was reprinted for the third time in 1815. Baillie, Hunter, Astley Cooper, Ferriar, Bateman, Luxmoore, and others, by their donations of books, amounting in all to about 160 volumes, laid the foundation of the present magnificent library. In these early days the Society was cramped for room, as the Council ordered that Mr. Nichols, the clerk, "be allowed to occupy the library when it is not otherwise wanted, and to procure a press bedstead at the Society's expense for his accommodation, to stand in the further corner of the meeting room." The Society moved to No. 3 Lincoln's Inn Fields in the year 1810, sharing the accommodation with the Geological Society, from whose president it borrowed £200 on the security of three Fellows. The two Societies did not separate till 1816. A temporary home was then found at 30, and afterwards at 57, Lincoln's Inn Fields, before the Society settled in 1834 at 53 Berners Street. The first meeting was held here on February 3rd, 1835, and the last on June 11th, 1889, when the Society moved into its present premises, No. 20 Hanover Square. An endeavour was made during the presidency of Sir Henry Halford in 1812 to obtain a Charter. The petition was signed and laid before the

Prince Regent in Council, but the proposition was so strongly opposed by the Royal College of Physicians that the prayer was not granted and it was not until 1834 that a second and more successful attempt was made to obtain a Royal Charter of Incorporation. The Society possesses a fine library of over 40,000 volumes which the Fellows can borrow at will; there is an extensive collection of engraved portraits of members of the medical profession, and the celebrated Chamberlen midwifery instruments are in its possession. In 1872 the Society undertook to administer the fund which had been raised to found a memorial to Dr. Marshall Hall. The memorial took the form of a prize to be given every five years for the best original work done during that period on the nervous system. The first award was made in 1878, Dr. Hughlings Jackson being the recipient, and the latest to Professor Sherrington. The Society, which had started originally as a protest against exclusiveness and monopoly, after a time fell into these very errors. Almost from the beginning the Society entirely discountenanced anything like discussion of a formal kind at its meetings, and any attempt to report its proceedings was rigorously suppressed, on the plea that the sale of the *Transactions* might be spoilt. Open dissatisfaction began to be expressed about 1840 at the manner in which the affairs of the Society were conducted. General practitioners were almost wholly excluded from the list of officers, and the manner in which the papers were selected for publication was very seriously criticised. Moreover, the attempt of some of the more energetic Fellows to introduce pathological specimens of cases for the purpose of discussion was not cordially received by the Council. These and the growing importance of the subject of pathology led to the formation of the Pathological Society of London.

### THE PATHOLOGICAL SOCIETY OF LONDON.

The Pathological Society of London owes its origin to Dr. Edward Bentley, who called a meeting at his house in Trinity Square, Borough, which was attended by eight gentlemen—Sir Richard Quain, Mr. James Moncrieff Arnott, Sir Risdon Bennett, Dr. Babington, Dr. Peacock, Dr. C. J. B. Williams and Dr. Barlow. After two or three meetings the Society was founded, and held its first meeting on October 20th, at 21 Regent Street. The objects of the Society were "the cultivation and promotion of pathology, by the exhibition and description of specimens, drawings, microscopic preparations, casts or models of morbid parts." Provision was made for the discussion of the specimens exhibited, but it was expressly enjoined upon the members that discussions upon abstract points should be avoided as far as possible, and that the topics of diagnosis and treatment should only be introduced in so far as was necessary to illustrate the pathology of the subject. These limitations clearly prove that the founders of the Society had no enlarged views as to a science of pathology, but were content with a study of morbid anatomy alone. Perhaps, however, they were not wholly wrong, for the science of pathology in 1846 was highly speculative, and abstract discussions would have been less profitable than the careful collection, comparison and record of individual specimens of disease. The result of the work of the Society has proved to be most valuable, and the issue of a well-edited and well-illustrated series of fifty volumes of *Transactions* has done much to advance the study of morbid anatomy in England. The Society moved in 1850 from 21 Regent Street, Waterloo Place, to 33 George Street, Hanover Square, and in 1857 it became a tenant of the Royal Medico-Chirurgical Society at 53 Berners Street, moving with it to the new premises in Hanover Square, where it holds most of its meetings at the present time. The seal of the Society is a well-executed head of Dr. Matthew Baillie [1761-1823] with the appropriate motto suggested by Dr. C. J. Williams, the first president, "Nec silet mors."

### THE MEDICAL RESEARCH CLUB.

The study of experimental pathology and the rise of bacteriology led to a much wider conception of pathology than had been held hitherto, and one for which the Pathological Society provided no accommodation. In 1891 forty of the younger working pathologists in Great Britain banded themselves into the Medical Research Club, which met without formality for the demonstration of original work in general and special pathological science. All communications made to the Society were absolutely private, as it was intended that members should bring forward their results for the friendly criticism and advice of their fellow members. The meetings were held in different laboratories, and any member who was absent from more than two meetings in the year ceased to be a member of the club. Many pleasant and instructive meetings were held, but the *raison d'être* of the club ceased in 1900, when the Pathological Society enlarged its scope and so widened its portals as to admit experimental pathologists. The club, therefore, is now in its *ætas capularis*.

### THE CLINICAL SOCIETY OF LONDON.

The Clinical Society of London owes its origin mainly to the energy of Dr. Greenhow and Sir J. Burdon Sanderson. The inaugural meeting was held at Dr. Greenhow's house in Queen Anne Street on October 29th, 1867, to consider the desirability of forming a society for the cultivation and promotion of practical medicine and surgery by the collection of cases of interest, especially of such as bear upon undetermined questions in pathology and therapeutics. A large number of members joined, and the Society began to hold its meetings in the rooms of the Royal Medical and Chirurgical Society at Berners Street, moving with its host to Hanover Square. The Clinical Society is best known perhaps from the work of its members in connection with the subject of Myxœdema. It publishes a series of *Transactions*, and has set a most excellent example to similar bodies by the compilation and publication of a single general index.

### THE OBSTETRICAL SOCIETY OF LONDON.

The inaugural meeting of the Obstetrical Society of London took place on December 16th, 1858, at the Freemasons' Tavern, when Dr. Rigby was in the chair and Dr. Tyler Smith was the moving spirit. In the course of the proceedings Dr. Granville, F.R.S., stated that a previous Obstetrical Society had been formed at his house as early as 1825, and that for a few years it had done good work in raising the status of that branch of the profession. He recalled the time, no further back than 1817, when anyone might practise midwifery without let or hindrance, and indeed without any medical qualification whatever. Things had then gone so far that a licence was sometimes taken by quacks to enable them to do their work with impunity, defying judge and jury when summoned before a court of law, by setting up as a defence that they did not pretend to be doctors, surgeons, or apothecaries, but only man-midwives. The degraded state of the art was such that the College of Physicians considered a licentiate practising midwifery as unworthy of a Fellowship; while a member of the College of Surgeons was deemed ineligible to be on the list of the Council or Court of Examiners if he practised as an accoucheur; and the Apothecaries' Company, which had been pressed to institute an examination in midwifery, long resisted "the soft persuasion." This being the case, the first Obstetrical Society, brought together in 1825, applied themselves to the removal of all such indignities, and to raise the practitioner in midwifery to a proper and dignified station. Sir Henry Halford, the President of the Royal College of Physicians of London, fought hard to perpetuate their exclusion from the College, saying that "midwifery was an unfit occupation for gentlemen of an academical education." But, after a struggle lasting for three years, the Society succeeded in obtaining the following concessions:—(1) A recognition of the honourable position of obstetricians amongst the medical practitioners of the three corporate bodies. (2) An examination in midwifery by the Apothecaries' Company. (3) The admission of persons practising midwifery (being members of the College of Surgeons) to be eligible for a post in the Council. (4) The declaration by the College of Physicians that licentiates practising midwifery should not be ineligible for the Fellowship of the College. These reforms cleared the way for the work of the present Obstetrical Society. Its labours have been mainly scientific, and its Fellows have thus done much to convert midwifery into scientific obstetric medicine, and have thereby still further raised their standing in the profession. It has not been altogether unmindful of its double purpose, however, for it has granted certificates of proficiency to midwives who have passed a qualifying examination before a committee of its Fellows.

The meetings of the Obstetrical Society have always been held in the house of the Royal Medical and Chirurgical Society of London, first in Berners Street and afterwards at Hanover Square. The Society has a good lending library for the use of its Fellows, and a museum of casts, models and instruments relating to the obstetric art. In 1868 the museum and library were lodged in rooms at 291 Regent Street, from which they were afterwards transferred to 54 Berners Street, but since 1882 they have been maintained at 20 Hanover Square.

### THE BRITISH GYNÆCOLOGICAL SOCIETY.

The British Gynæcological Society was founded on December 27th, 1884, at a meeting held in the rooms of the Medical Society of London, Dr. Routh being called to the chair. The meeting was the outcome of a feeling that the Obstetrical Society was unable to devote a sufficient time at its meetings to the important subject of Gynæcology, since the work of Clay, Simpson, Tait, Matthews Duncan and others has borne abundant fruit of late years, and has led to a well-recognised surgical treatment of many of the diseases peculiar to women. The Society at once began active work, and since 1885 it has published its proceedings as *The British Gynæcological Journal*. The meetings are held at 20 Hanover Square, and from time to time in the large towns of Great Britain and Ireland.

It may be considered fairly that the societies hitherto mentioned are the offspring—direct or indirect—of the Medical Society of London. The remaining societies are of independent origin.

### THE HUNTERIAN SOCIETY.

Mr. Armiger, Assistant Surgeon to the London Hospital, first originated the idea of founding a medical society in the East of London. His design was carried into effect by Dr. Cooke, of Great Prescott Street, who called a meeting at his house on November 11th, 1818, the Society being inaugurated at the King's Head, in the Poultry, on January 20th, 1819, when Sir William Blizard was elected the first president and Dr. Conquest and Mr. Armiger the first secretaries. The society met at first in the rooms of the London Orphan Society, in St. Mary Axe; it moved in 1821 to 18 Aldermanbury, and in 1834 to 4 Blomfield Street. Here the Society remained until 1866, when the Council secured accommodation at the London Institution in Finsbury Circus. The Society has a library, issues reports, and affords an annual oration.

### THE HARVEIAN SOCIETY.

The Harveian Society of London was established at the Western General Dispensary in Lisson Grove on September 12th, 1831, for the convenience and improvement of members of the medical profession who practised in the western part of London. It was founded as the West London Medical Society, but almost immediately changed its name to the Harveian Society of London, at the same time moving to 28 Edward Street (now Seymour Street), Portman Square. In 1835 the Society moved to the Marylebone Literary and Scientific Institution in Edward Street, but for many years past it has found accommodation at the Stafford Rooms, in Titchborne Street, Edgware Road. The Harveian lectureship, consisting of two or three lectures, was founded in 1875, and has been productive of several interesting courses by distinguished members of the profession.

### THE WEST LONDON MEDICO-CHIRURGICAL SOCIETY.

This Society was founded in 1882 at a meeting held in the board room of the West London Hospital at Hammersmith, and under able management has proved itself a most useful and successful body. The society issues annually a volume of *Proceedings*, and maintains the Cavendish lectureship, called after Henry Cavendish, who lived for some time near the spot where the Society meets.

These societies may be looked upon as the General Medical Societies of London. The remainder have been formed with the avowed object of promoting intercourse amongst those who are practising special branches of the profession. An attempt, therefore, has usually been made to give them a national rather than a local importance, and though all the societies hold most of their meetings in London, many make it a rule to meet from time to time in the different chief provincial towns.

### THE MEDICO-PSYCHOLOGICAL ASSOCIATION.

The cares of a lunacy practice seem to have first banded specialists together into a society. The Medico-Psychological Association was established in 1841 for the improvement of asylums and hospitals for the insane—the acquisition and diffusion of a more extended knowledge of insanity and its treatment, and the promotion of a free communication on such subjects between its members. The Association consists of medical officers of hospitals and asylums for the insane (public as well as private), and of legally qualified practitioners otherwise engaged in or interested in the treatment of insanity. The meetings are held at the rooms of the Medical Society of London, though for some years the Association met annually at some private or public asylum. In 1852 the proceedings were published as *The Asylum Journal of Mental Science,* under the editorship of Dr. Bucknill, which became in 1858 *The Journal of Mental Science.* The Association was incorporated in 1895.

### THE ODONTOLOGICAL SOCIETY OF LONDON.

The dentists also associated themselves early into a special society. An attempt was made in 1841 to obtain public recognition for dentistry as a branch of surgery, but it was not successful until 1855, and in the following year the Odontological Society was formed. The object of the Society was to obtain a recognised qualification for dentists from the Royal College of Surgeons of England. About the same time the College of Dentists of England was founded as an independent organisation, whose promoters desired that dentistry should be free from any association with existing licensing bodies. The College of Dentists examined candidates and granted licences, but it only lived seven years. It amalgamated with the Odontological Society in 1863, the new body being known as the Odontological Society of Great Britain. The Society has a library and a museum, and has published *Transactions* regularly since 1858.

### THE EPIDEMIOLOGICAL SOCIETY OF LONDON.

The Epidemiological Society of London was founded in 1850, at the suggestion of Mr. J. H. Tucker, to investigate epidemic or spreading diseases. The inaugural meeting was held on July 30th at Hanover Square Rooms under the presidency of Lord Ashley (afterwards the Earl of Shaftesbury). The monthly meetings were held at first at the house of the Royal Medical Benevolent College, 37 Soho Square, but of late years they have been transferred to the premises of the Medical Society of London in Chandos Street, Cavendish Square. The Society has always done most excellent work, though it has ranked amongst the more select and aristocratic bodies in the profession.

### THE POOR-LAW MEDICAL OFFICERS' ASSOCIATION OF ENGLAND AND WALES.

The Association was founded in 1868, by the union of the Poor-Law Medical Reform Society and the Metropolitan Poor-Law Medical Officers' Association, to obtain life appointment and adequate remuneration for all Poor-Law Medical Officers in England and Wales: for mutual assistance and to provide a channel through which all defects in the Poor-Law Medical Service may be brought to light and discussed with a view to their removal or amelioration. The Council meet at 9 Copthall Avenue, and there is an annual general meeting of the Association for the election of officers. A similar society is urgently needed at the present time in Ireland, where some of the local authorities in the more remote districts are attempting to ride roughshod over their medical officers.

### THE SOCIETY OF MEDICAL OFFICERS OF HEALTH.

The Association of Medical Officers of Health was founded in 1856, with Sir John Simon as the first president. It assumed its present title in 1873. Annual Reports of the proceedings were published from 1868-79, and *Transactions* from 1880-7, but since 1888 *Public Health* has been the organ of the Association. The meetings are held at 197 High Holborn, W.C.

### THE OPHTHALMOLOGICAL SOCIETY FOR GREAT BRITAIN AND IRELAND.

The Ophthalmological Society of the United Kingdom was founded at the rooms of the Medical Society of London on June 23rd, 1880, when the chair was occupied by (Sir) William Bowman. From the outset the Society took the wise course of resisting the tendency to specialism by enrolling amongst its members a number of physicians who took an interest in diseases of the eye in their medical aspects, and this admixture has given a peculiar and very beneficial character to the Society. The Ophthalmological Society meets at the house of the Medical Society of London; it publishes *Transactions,* and supports a special Bowman Lecture, given annually by some distinguished home or foreign ophthalmic surgeon, in honour of the first President, Sir William Bowman, a founder of modern ophthalmic surgery.

### THE NEUROLOGICAL SOCIETY OF LONDON.

The Neurological Society of London was founded on November 14th, 1885. The meetings are held at the rooms of the Medical Society at irregular intervals, and its highly valuable proceedings are published in *Brain.*

### THE ROYAL INSTITUTE OF PUBLIC HEALTH.

The Institute of Public Health was founded in 1886 to provide for the association of medical practitioners engaged in Public Health, State Medicine, and Sanitary Science; to aid the theoretical and practical investigation and study of all branches of Public Health medicine by holding meetings, instituting lectures, and issuing publications. The Association examines candidates and issues certificates of proficiency to sanitary inspectors, and it has formed branches in Scotland and Ireland. The official organ of the Association is the *Journal of State Medicine,* published monthly. The meetings are held at 197 High Holborn. The Society was incorporated in 1892.

### THE SANITARY INSTITUTE.

The objects of the Institute are to promote the advancement of sanitary science in all or any of its branches, and to diffuse knowledge relating thereto. Examinations are held for inspectors of nuisances and for meat inspectors, as well as for school teachers, and certificates of proficiency are awarded. The Institute also teaches, and the Parkes Museum is incorporated with it at Margaret Street, Cavendish Square.

### THE ANATOMICAL SOCIETY OF GREAT BRITAIN AND IRELAND.

The foundation meeting of the Anatomical Society, established by the activity of Mr. C. B. Lockwood, was held at the rooms of the Medical Society of London on Friday, May 6th, 1887, Sir George Murray Humphry being in the chair. The scope of the Society was declared to be the anatomy, embryology and histology of man and of animals in so far as they throw light upon the structure of man. The meetings are held quarterly at the Anatomical Departments of the various Universities and medical schools of the United Kingdom, the communications being published in the *Journal of Anatomy*.

### THE DERMATOLOGICAL SOCIETY OF LONDON.

The Dermatological Society of London meets at the rooms of the Medical Society in Chandos Street, W. It was founded in 1882.

### THE DERMATOLOGICAL SOCIETY OF GREAT BRITAIN AND IRELAND.

This Society was founded in 1894, and holds its meetings at 20 Hanover Square.

### THE LARYNGOLOGICAL SOCIETY OF LONDON.

The Laryngological Society of London was established on February 13th, 1893, for the cultivation and promotion of laryngology, rhinology and the allied sciences. Its meetings are held at 20 Hanover Square.

### THE SOCIETY OF ANÆSTHETISTS.

The Society of Anæsthetists meets at 20 Hanover Square, and was founded in 1893.

### THE BRITISH BALNEOLOGICAL AND CLIMATOLOGICAL SOCIETY.

The British Balneological and Climatological Society was founded at a meeting of medical practitioners connected with British health resorts. The meeting was held at Limmer's Hotel, Conduit Street, W., on November 9th, 1895, when it was determined to provide means for the association of medical practitioners connected with British health resorts, and to promote good fellowship amongst themselves and with members of other branches of the medical profession; to encourage the theoretical and practical investigation and study of balneotherapeutics and medical climatology; and to advocate and sustain within the profession the interests and claims of British balneology and climatology. Sir Edward Sieveking was elected the first president. The meetings are held in the rooms of the Royal Medical and Chirurgical Society of London on Wednesdays at dates fixed by the Council of the Society, and the *Proceedings* are issued in a journal.

The older societies have a local habitation as well as a name. They are governed by a hierarchy and the meetings are formal in character. The younger societies have in many cases a name only, for they are migratory, their government is democratic, and their meetings are more or less informal.

### THE PHYSIOLOGICAL SOCIETY.

The earliest of the latter-day societies was the Physiological Society, established soon after the passing of the Vivisection Act in 1876, as a dining club of fifty working physiologists. It is governed by a committee, a secretary and a treasurer, the chairman being chosen for the evening at each meeting. The work of the Society has increased enormously, and its meetings are held at the various physiological laboratories in London, Oxford and Cambridge. The number of members is unlimited, and the dinner, which was at first an essential feature, is now quite subordinate. The *Proceedings* of the Society are published in the *Journal of Physiology*, of which a copy is distributed to every member.

### THE BRITISH ORTHOPÆDIC SOCIETY.

The British Orthopædic Society was founded at Bristol on August 3rd, 1894, during the annual meeting of the British Medical Association. It was laid down somewhat upon the lines of the Physiological Society, except that there was no dinner. The meetings are held during the winter months at the various orthopædic hospitals in London and the chief towns in the provinces. It affords many opportunities for surgeons interested in orthopædic practice to meet together and exchange opinions on subjects associated. It publishes a volume of *Transactions* every year.

### THE SOCIETY FOR THE STUDY OF DISEASE IN CHILDREN.

The Children's Society is the youngest of the medical societies, for though it is founded it has not yet held more than a preliminary meeting. It is to meet at the different children's hospitals in London or elsewhere, and, like the Physiological and the British Orthopædic Societies, is to be presided over by a chairman selected for the occasion. The treasurer is the only permanent officer.

